# Liposome-Encapsulated Bioactive Guttiferone E Exhibits Anti-Inflammatory Effect in Lipopolysaccharide-Stimulated MH-S Macrophages and Cytotoxicity against Human Cancer Cells

**DOI:** 10.1155/2022/8886087

**Published:** 2022-08-30

**Authors:** Jean Paul Dzoyem, Shashank Reddy Pinnapireddy, Hugues Fouotsa, Jana Brüßler, Frank Runkel, Udo Bakowsky

**Affiliations:** ^1^Department of Biochemistry, Faculty of Science, University of Dschang, P.O. Box 67, Dschang, Cameroon; ^2^Department of Pharmaceutics and Biopharmaceutics, University of Marburg, Robert-Koch-Str. 4, 35037 Marburg, Germany; ^3^CSL Behring GmbH, Emil-von-Behring-Str. 76, 35041 Marburg, Germany; ^4^Department of Engineering Process, National Higher School, University of Douala, P.O. Box 2701, Douala, Cameroon; ^5^Institute of Bioprocess Engineering and Pharmaceutical Technology, University of Applied Sciences Mittelhessen, Wiesenstrasse 14, 35390 Giessen, Germany; ^6^Faculty of Biology and Chemistry, Justus-Liebig University Giessen, Heinrich-Buff-Ring 17, 35392 Giessen, Germany

## Abstract

**Background:**

Guttiferone E is a naturally occurring polyisoprenylated benzophenone exhibiting a wide range of remarkable biological activities. But its therapeutic application is still limited due to its poor water solubility. This study is aimed at preparing guttiferone E-loaded liposomes and assessing their *in vitro* cytotoxicity and anti-inflammatory effect.

**Methods:**

Liposomes containing guttiferone E were prepared by the thin film hydration method, and the physicochemical characteristics were determined using dynamic light scattering, laser Doppler velocimetry, and atomic force microscopy. The cytotoxicity was assessed by the MTT assay. The fluorometric cyclooxygenase (COX) activity assay kit was used to assess the COX activity while the nitric oxide production was evaluated by the Griess reagent method.

**Results:**

The liposomes with a mean size of 183.33 ± 17.28 nm were obtained with an entrapment efficiency of 63.86%. Guttiferone E-loaded liposomes successfully decreased the viability of cancer cells. The overall IC_50_ values varied between 5.46 *μ*g/mL and 22.25 *μ*g/mL. Compared to the untreated control, guttiferone E-loaded liposomes significantly reduced the nitric oxide production and the activity of COX in a concentration-dependent manner.

**Conclusion:**

This study indicates that liposomes can be an alternative to overcome the water insolubility issue of the bioactive guttiferone E.

## 1. Introduction

Inflammation has long been recognized as a hallmark of the development of cancer. It is now becoming clear that mediators and cellular effectors of inflammation are important constituents of the tumor microenvironment [1, 2]. Nonsteroidal anti-inflammatory drugs (NSAIDs) and chemotherapy continue to be the first-line treatment option for most inflammatory conditions and cancers [3, 4]. However, these treatments cause undesirable side effects including severe toxicity to the healthy tissues. Therefore, searching for new therapeutic agents for the treatment of inflammation and cancers is needed. Natural products are an important source of therapeutic agents. In the past few years, several studies have investigated the anti-inflammatory and anticancer potential of plant-derived products, and many bioactive molecules were isolated and identified [5]. The cyclooxygenase inhibitory activity of many active ingredients derived from plants is well established in the literature. These include coumarins, alkaloids, flavonoids, cinnamates, stilbenes, and xanthones [6]. Several examples from the literature show the potential of naturally occurring compounds to act as cyclooxygenase inhibitors and prevent various types of cancers [7].

**Figure 1:**
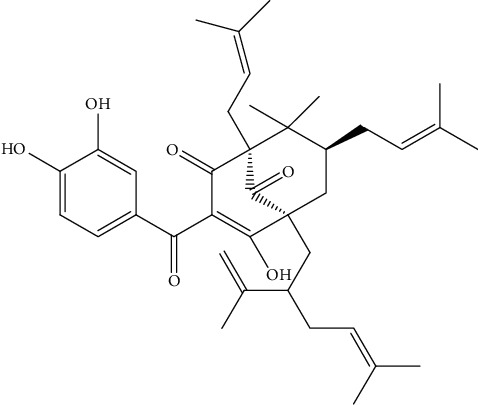
Chemical structure of guttiferone E.

Guttiferone E (GE) is a naturally occurring polyisoprenylated benzophenone usually found in plants of the Clusiaceae family (Figure 1). GE has been reported to exert a wide range of biological activities including anti-inflammatory and cytotoxic effects against cancer cells [8]. Its anti-inflammatory mechanism includes the targeting of the TLR/IRAK-1 pathway and inhibition of the downstream NF-*κ*B and Akt/mTOR signaling pathways and the inhibition of the activity of lipoxygenase enzymes and the production of nitric oxide in LPS-stimulated RAW 264.7 macrophages [9–11]. GE has been reported to display cytotoxic activity in HCT 116, HT 29, and SW-480 human colon cancer cell lines, in the KB oral carcinoma cell line, and in various sensitive and drug-resistant phenotype cancer cells including breast adenocarcinoma MDA-MB-231-BCRP cells [12–14]. The cytotoxic mode of action of guttiferone E in cancer cells encompasses the interference with mitochondrial membrane potential, increase in the expression of genes such as XBP1, ATF4, and DDIT3/CHOP, and the subsequent activation of the endoplasmic reticulum stress/apoptotic pathway as well as the activation of apoptotic enzymes caspase 3/7, caspase 8, and caspase 9 [12, 13]. Despite the remarkable pharmacological potential of guttiferone E, its therapeutic application is still very limited due to its poor aqueous solubility. Because of this drawback, this promising compound cannot move forward in the drug discovery pipeline to clinical trials. These limitations could be overcome by encapsulating guttiferone E with suitable drug delivery nanomaterials. As the main components of the cellular membrane, phospholipids have excellent biocompatibility and their amphiphilic structure confers a propensity to form liposomes, which can be smartly employed as drug carriers [15]. Among various drug delivery carriers, liposomes are the most common and well-investigated nanocarriers. Liposomes have been used as a drug delivery carrier for a wide range of therapeutic compounds including bioactive natural products. Moreover, liposomes consist of an aqueous core surrounded by a lipid bilayer. They can carry both hydrophobic and hydrophilic drugs and molecules to a target site [16]. Successful therapeutic potential enhancement of some liposome-loaded natural plant products such as curcumin and *α*-mangostin has been reported [16, 17]. There is a hope that overcoming the guttiferone E water insolubility issue might improve its efficacy and provide an effective therapeutic agent for future clinical use. In this regard, this study was undertaken to prepare GE liposomes and evaluate their physicochemical properties. Then, compare the cytotoxicity of GE-loaded liposomes to that of free guttiferone E on various human cancer cells. In addition, the ability of guttiferone E liposomes to inhibit the production of nitric oxide as well as the activity of cyclooxygenase enzymes in LPS-stimulated MH-S murine macrophages was investigated.

## 2. Materials and Methods

### 2.1. Natural Compounds and Chemicals

Guttiferone E was isolated from the stem bark of *Garcinia punctata*; the isolation procedure and the structure elucidation including necessary analytical techniques for compound purity were previously described [10]. Dipalmitoylphosphatidylcholine (DPPC) and cholesterol (CHOL) were purchased from LIPOID, Germany. Penicillin/streptomycin/fungizone (PSF), fetal bovine serum (FBS), culture media, and [3-(4.5-dimethylthiazol-2-yl)-2.5-diphenyltetrazolium bromide] (MTT) were supplied by Capricorn Scientific, Germany. Lipopolysaccharide (LPS) (*Escherichia coli* 0111:B4), sodium nitrite, the Griess reagent, and curcumin (from *Curcuma longa*) were provided by Sigma, Germany. Lysis buffer was obtained from Promega, Germany.

### 2.2. Preparation of Guttiferone E Liposomes

Guttiferone E-loaded liposomes (GEL) with a lipid weight ratio of 1 : 5 were prepared by the thin film hydration method as reported [18]. Briefly, the lipid phase containing DPPC and CHOL to a molar ratio of 7 : 3 was dissolved in chloroform and methanol mixture (2 : 1 *v*/*v*), and guttiferone E was dissolved in ethanol. The solutions were mixed, and the organic solvents were removed by rotary evaporation (Heidolph Laborata 4000 Efficient) to obtain a thin lipid film. The thin lipid mixture film was hydrated with phosphate-buffered saline (PBS pH 7.4) and agitated by hand shaking and further bath-sonicated in an ultrasonic bath (Elmasonic P) at 41°C for 10 minutes to obtain guttiferone E liposomes.

### 2.3. Characterization of the Liposomes

The physicochemical characteristics of the prepared guttiferone E-loaded liposomes were determined by dynamic light scattering (DLS) and laser Doppler velocimetry using a Zetasizer Nano ZS (Malvern Instruments, Herrenberg, Germany) equipped with 10 mW HeNe laser at a wavelength of 633 nm at 25°C. Laser attenuation and measurement position were adjusted automatically by the instrument. The zeta potential was measured via electrophoretic mobility with laser Doppler velocimetry. The average values of the size intensity peak and zeta potential were calculated with data of three independent experiments ± standard deviation. Each sample was measured three times with at least 10 subruns. Parameters measured included size (hydrodynamic diameter), zeta potential, and the polydispersity index. The morphology was observed using atomic force microscopy.

### 2.4. Atomic Force Microscopy (AFM)

AFM was used to determine the morphology of the particles and to confirm the particle size as previously described [19]. A small amount of the sample (10 *μ*L) to be analyzed was pipetted onto silica wafers which were glued to glass slides. The samples were allowed to dry before being gently washed with water followed by nitrogen gas. Surface analysis was performed under ambient conditions using a Nanowizard® 3 Nanoscience AFM (JPK Instruments, Berlin, Germany) using intermittent contact mode (tapping mode) with an aluminum coated silicon nitride probe (HQ: NSC14/Al BS having a 160 kHz resonance frequency and a force constant of 5 N/m) from *μ*masch (Tallinn, Estonia) at scan rates between 0.5 and 1 Hz. The raw images were processed using JPKSPM data processing software (JPK Instruments).

### 2.5. Guttiferone E Encapsulation Efficiency (EE)

Encapsulation efficiency was calculated as described by Elmi et al. [20] with slight modifications. Briefly, liposomes were centrifuged at 15000 × *g* for 15 min at 4°C. The supernatant was collected, frozen at -80 for 24 h, and lyophilized (Alpha 1-4 LCS Christ lyophilizer). Then, the obtained powder was dissolved in ethanol, and appropriate dilution was made to measure the guttiferone E content using a spectrophotometer (FLUOstar OPTIMA) at 307 nm. This wavelength was selected from the preliminary photometric reading of free GE between 200 and 800 nm. The readings were applied to a calibration curve made with guttiferone E. Encapsulation efficiency (EE) was calculated using the following formula: %EE = [(Ci–Cf)/Ci] × 100, where Ci is the concentration of the initial guttiferone E added and Cf is the concentration of free guttiferone E recovered in the supernatant.

### 2.6. Cytotoxicity of Guttiferone E-Loaded Liposomes on Human Cancer Cell Lines

#### 2.6.1. Cell Lines and Culture

The effect of GEL on cell growth was evaluated in a panel of human tumor cells belonging to various organs. These included lung A549 adenocarcinoma, human breast carcinoma cell SKBr-3, human ovarian carcinoma SKOV-3, and human T cell leukaemia cells JURKAT obtained from American Type Culture Collection (ATCC). Human epithelial colorectal adenocarcinoma cells Caco-2 were courtesy of Prof. Dr. Frank Runkel from the Institute of Biopharmaceutical Technology, University of Applied Sciences Giessen, Germany. Murine alveolar macrophage cells (MH-S) obtained from the European Collection of Cell Cultures (ECACC) were used for anti-inflammatory activity. SKOV-3 and SKBr-3 were cultured in Iscove's Modified Dulbecco's Medium (IMDM). Caco-2 and A549 were cultured on DMEM (Dulbecco's Modified Eagle Medium) high glucose with sodium pyruvate while JURKAT and MH-S were maintained on RPMI containing *β*-mercaptoethanol 0.05 mM. All media were supplemented with 10% fetal bovine serum (FBS) and 1% penicillin/streptomycin/fungizone (PSF) solution. The cell lines were cultured at 37°C in a humidified environment containing 5% CO_2_.

#### 2.6.2. MTT Assay

*In vitro* cytotoxicity against above-mentioned cell lines was performed by the [3-(4.5-dimethylthiazol-2-yl)-2.5-diphenyltetrazolium bromide] MTT assay. Briefly, cells were harvested in the log phase using trypsin (0.05% trypsin and 0.02% EDTA in PBS). The cell suspensions were diluted with an appropriate growth medium to obtain the cell density of 10^4^ cells/well. Aliquots of 100 *μ*L of each suspension were seeded in 96 wells of cell culture plates. The cells were incubated at 37°C in an atmosphere of 5% CO_2_ and 95% relative humidity in a CO_2_ incubator. After 24 h incubation, guttiferone E-loaded liposomes and free guttiferone E at varying concentrations were added to the wells containing cells. For free GE, the final concentration of DMSO was 0.2% which did not affect the cell viability. Suitable controls with blank liposomes or equivalent concentrations of DMSO were also included. Doxorubicin was used as a reference cytotoxic drug. The plates were further incubated for 48 h; then, the medium in each well was aspirated and MTT solution (2 mg/mL in PBS) was diluted to 1 : 10 with fresh medium and added to each well, and the plates were further incubated for 4 h. The medium was aspirated from the wells, and DMSO was added to solubilize the formed formazan crystals. The absorbance was measured on a FLUOstar OPTIMA microplate reader at 570 nm. The concentration causing 50% inhibition of cell growth (IC_50_) was calculated from the concentration-inhibition response curve by regression analysis.

### 2.7. Intracellular Guttiferone E-Loaded Liposome Uptake Study

Liposomes labeled with rhodamine-DPPE were used to investigate the cellular uptake in MH-S cells. Cells (90,000 per well) were seeded onto 12-well plates (Nunclon Delta, Nunc GmbH & Co. KG., Wiesbaden, Germany) containing coverslips (15 mm diameter). The plates were incubated for 24 h before being used for the studies. 30 *μ*g and 50 *μ*g of the liposomes were added into wells in triplicate; the plates were gently swirled and incubated for 24 h. The cells were then washed twice with PBS containing Ca^+^ and Mg^+^ and fixed with 4% formaldehyde solution for 20 min after which the cell nucleus was counterstained with DAPI (4,6-diamidino-2-phenylindole) for 20 min. Finally, the cells were washed with PBS, and the coverslips were mounted onto slides and sealed. The cells were examined under a confocal laser scanning microscope (CLSM) (Zeiss Axiovert 100 M, Carl Zeiss Microscopy GmbH, Jena, Germany). An argon ion laser (Coherent Enterprise, Coherent Inc., California, USA) with 364 and 543 nm wavelengths for observing nuclear counterstaining and Rhodamine B-labeled liposomes, respectively, was used. A detector equipped with a 585 nm long-pass filter for Rhodamine B-labeled liposome and 385 nm long-pass filter for DAPI was used for recording the micrographs.

### 2.8. Anti-Inflammatory Activity

#### 2.8.1. Effect of Guttiferone E-Loaded Liposomes on the Nitric Oxide (NO) Production in LPS-Stimulated MH-S Macrophages

The murine macrophage cells MH-S (10^4^ cells/well) were seeded in 96-well plates and incubated for 24 h. Then, cells were treated with guttiferone E-loaded liposomes or curcumin at different concentrations and/or LPS (0.1 *μ*g/mL) (*Escherichia coli* 0111:B4) and incubated further for 24 h. Thereafter, supernatants were collected, and the organic nitrite concentration was measured as an indicator of NO production using the Griess reagent. Briefly, 100 *μ*L of cell culture supernatant was mixed with the equal volume of the Griess reagent and incubated at room temperature for 10 min, and then, the absorbance at 550 nm was measured on a FLUOstar OPTIMA microplate reader. A fresh culture medium was used as the blank in all experiments. Nitrite concentration was determined by interpolation of standard curves constructed with known concentrations of NaNO_2_, and the percentage of inhibition was determined relative to the control and therefore the IC_50_ values.

To ascertain that the nitric oxide inhibition observed was not due to the cytotoxic effect of the sample, an MTT assay was performed on the cells after the collection of the supernatant. The percentage of cell viability was calculated as described above for the cytotoxicity assay.

#### 2.8.2. Effect of Guttiferone E-Loaded Liposomes on the Activity of Cyclooxygenases 1 and 2 in LPS-Stimulated MH-S Macrophage Lysate

*(1) Cell Lysate Preparation*. MH-S cells were seeded at 10^6^ cells/mL in a 48-well microplate and allowed to adhere for 24 h and then treated with guttiferone E-loaded liposomes or diclofenac at a different concentration as well as LPS 1 *μ*g/mL. After 24 h, cells were detached with TNE buffer (Tris 40 mM, NaCl 150 mM, and EDTA 1 mM) and washed with PBS (1x), then resuspended in 1 mL PBS (1x), transferred into a 1.5 mL tube, and centrifuged at 500 × *g* for 3 min. The pellet was then resuspended in 0.5 mL of lysis buffer with a protease inhibitor cocktail, vortexed, and incubated in the ice for 5 min. The obtained cell lysate was centrifuged at 12000 × g for 3 min, and the supernatant was collected and kept on the ice for the COX activity assay.

*(2) COX Activity Assay*. The fluorometric cyclooxygenase (COX) activity assay kit (Biovision) was used according to the manufacturer's instructions to examine the ability of the GEL to inhibit the COX-1/COX-2 isozyme activity. The assay includes COX-1 and COX-2 specific inhibitors to differentiate the activity of COX-1 and COX-2 as well as other peroxidases. Diclofenac sodium salt (Cayman Chemical Company) 5 *μ*M was used as a standard drug, and appropriate controls were also included.

### 2.9. Statistical Analysis

The values expressed are means of three replicate determinations ± standard deviation. A pairing comparison between free guttiferone and guttiferone E-loaded liposomes was carried out by analysis of variance (ANOVA) using Dunnett's multiple comparisons test.

## 3. Results

The incorporation of nanoparticles into a delivery system for poorly water-soluble bioactive natural products is a major advance in the efforts to increase their therapeutic effects. In this study, the naturally occurring guttiferone E (Figure 1) was encapsulated into phospholipids as a carrier to overcome its poorly water solubility issue and improve its pharmacological properties. The physicochemical properties of the formulated liposomes were determined, and so was their anti-inflammatory effect as well as their effect on the proliferation of human cancer cell lines.

### 3.1. Characteristics of Guttiferone E-Loaded Liposomes

**Figure 2:**
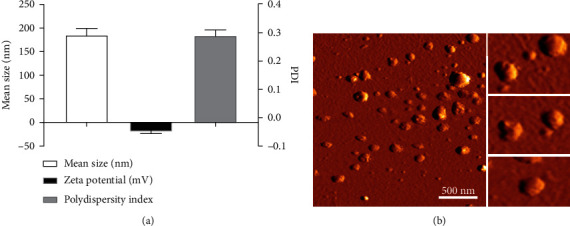
(a) Characteristics of the guttiferone E-loaded liposomes. (b) Atomic force microscopy (AFM) images of the size distribution and morphology of guttiferone E-loaded liposomes.

Liposomes composed of dipalmitoylphosphatidylcholine (DPPC) and cholesterol were prepared by the thin film hydration method. DLS data showed that the mean diameter of liposome nanoparticles was 183.33 ± 17.28 nm with a polydispersity index of 0.30 ± 0.01 (Figure 2(a)). The liposome size as observed by AFM (Figure 2(b)) correlated well with the size measured by DLS. The zeta potential of the guttiferone E-loaded liposomes was −12.43 ± 1.52. The zeta potential of the liposomes was negative indicating the stability of the liposome suspension without the tendency of aggregation.

### 3.2. Cytotoxicity of Guttiferone E-Loaded Liposomes on Human Cancer Cell Lines

**Figure 3:**
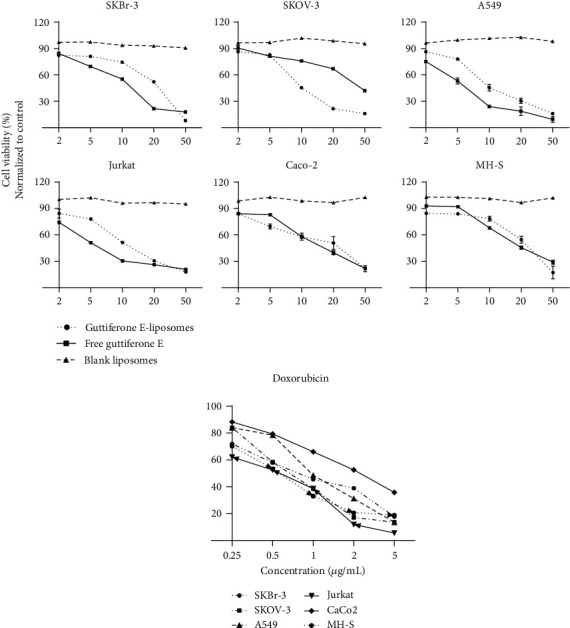
Guttiferone E-loaded liposome inhibition of the growth of human cancer cell lines. Cell viability (MTT) assays were performed using equivalent dosages of free guttiferone E dissolved in DMSO, guttiferone E-loaded liposomes, and blank liposomes in a panel of human cancer cell lines.

Guttiferone E-loaded liposomes were tested for their cytotoxic activity against five human cancer cell lines, and the results were compared with those of free guttiferone E dissolved in DMSO. As shown in Figure 3, guttiferone E-loaded liposomes successfully decreased the cell viability of cancer cells with a percentage of cell death of at least 75% in all the cell lines used. However, compared to free guttiferone E, liposomes selectively reduced the viability of cancer cells.

In a preliminary experiment, guttiferone E and guttiferone E-loaded liposomes were tested at 50 *μ*g/mL and both exhibited more than 50% inhibition on a panel of human cancer cell growth. Therefore, they were further tested at different concentrations and then the corresponding IC_50_ values. Results are presented in Table 1.

The IC_50_ values of guttiferone E-loaded liposomes were found to be between 9.73 *μ*g/mL and 22.55 *μ*g/mL while that of free guttiferone E (dissolved in DMSO) ranged from 5.46 *μ*g/mL to 40.18 *μ*g/mL among all the cancer cell lines. Blank liposomes did not show any activity. An interesting cytotoxic effect of guttiferone E-loaded liposomes was observed against Skov-3 and Jurkat with respective IC_50_ values of 9.73 *μ*g/mL and 10.40 *μ*g/mL. Unlike all other cancer cell lines used in this study, guttiferone E-loaded liposomes were more effective in inhibiting the proliferation of human ovarian carcinoma SKOV-3 (IC_50_ value of 9.73 *μ*g/mL) as compared to that shown by free guttiferone E (IC_50_ value of 40.18 *μ*g/mL), by increasing the cytotoxicity of liposomes by 4.1-fold.

### 3.3. Intracellular Guttiferone E-Loaded Liposome Uptake

**Figure 4:**
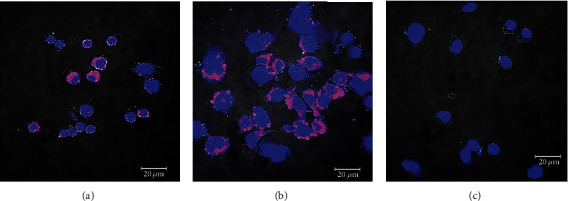
Cellular uptake of guttiferone E-loaded liposomes by MH-S cells examined under a CLSM. MH-S cells in culture were incubated for 24 h at 37°C with 30 *μ*g (a) and 50 *μ*g (b) rhodamine-DPPE-labeled liposomes. (c) Control. Blue and red colors correspond to the nucleus and liposomes, respectively.

Cellular uptake of liposomes was performed in MH-S macrophage cells using DPPE-labeled rhodamine. Confocal laser scanning microscope images are shown in Figure 4. It can be seen from the images that the liposomes are inside the cells and the number of liposomes labeled with rhodamine-DPPE internalized increased with the concentration. A bright-field channel was used to visualize the cell boundaries.

### 3.4. Effect of GEL on the Nitric Oxide (NO) Production by Guttiferone E-Loaded Liposomes in LPS-Stimulated MH-S Macrophages

**Figure 5:**
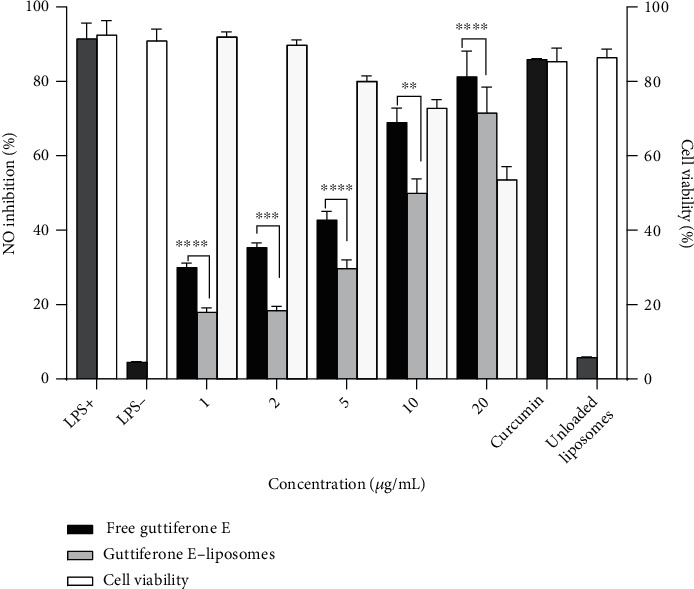
Effect of the guttiferone E-loaded liposomes on the nitric oxide production in LPS-stimulated macrophages compared to free GE. MH-S cells were incubated with the indicated concentrations of guttiferone E and guttiferone E-loaded liposomes, for 24 h in the presence (+) or absence (-) of LPS; then, from the supernatant, NO was detected and analyzed using the Griess reagent. Curcumin (Cur) at 5 *μ*g/mL was used as a positive control and unloaded liposomes as a vehicle control at 20 *μ*g/mL. Data are the mean from three independent experiments. Dunnett's multiple comparisons test using two-way ANOVA was performed: ^∗^*p* < 0.01, ^∗∗∗^*p* < 0.001, and ^∗∗∗∗^*p* < 0.0001 for free guttiferone E versus guttiferone E-loaded liposomes.

MH-S murine alveolar macrophages were treated with different concentrations of guttiferone E and guttiferone E-loaded liposomes and stimulated with LPS 1 *μ*g/mL; then, the amount of NO release was measured as well as the cell viability. To exclude the possibility that the observed anti-inflammatory effects could be due to cytotoxicity, samples were used only at concentrations in which cell proliferation was not affected according to the calculated IC_50_. Curcumin, a well-known inhibitor of nitric oxide production, was used as a reference compound at a single concentration of 5 *μ*g/mL [21]. Results are presented in Figure 5.

Our data demonstrate that both free guttiferone E and guttiferone E-loaded liposomes inhibited NO production in a concentration-dependent manner (Figure 5). The percentages of NO production inhibition were 17.99%, 18.37%, 29.73%, 49.95%, and 71.55% by addition of 1, 2, 5, 10, and 20 *μ*g/mL of guttiferone E-loaded liposomes, while they were 29.99%, 35.37%, 42.73%, 68.95%, and 81.25% for free guttiferone E. Curcumin at a single concentration of 5 *μ*g/mL showed an inhibition percentage of 85.88%. Subsequently, we evaluated the effect of free and guttiferone E-loaded liposomes on MH-S viability by performing the MTT assay. Our results showed that they did not exert any significant effect on MH-S viability; the average of their percentage of cell growth inhibition was 53.55% (Figure 5). This observation was expected since only concentrations at which cell proliferation is not significantly affected were used. In our previous study on RAW 264.7 cells, we reported a percentage of NO production inhibition of 90% for free guttiferone E at 6.25 *μ*g/mL. However, this inhibition was also associated with a more toxic effect against RAW 264.7 macrophages with a percentage of cell viability of 35.31% [10].

### 3.5. Effect of GEL on the Activity of Cyclooxygenases 1 and 2 in LPS-Stimulated MH-S Macrophage Lysate

**Figure 6:**
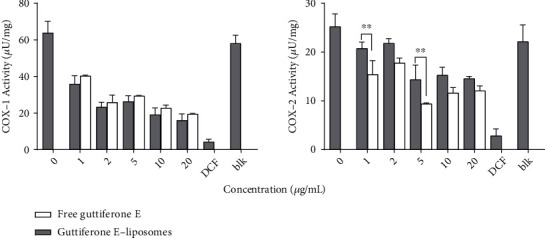
Detection of COX-1 and COX-2 activity in LPS-stimulated MH-S cell lysate treated with different concentrations of free guttiferone E and guttiferone E-liposomes. The positive control diclofenac (DCF) was used at 5 *μ*M, and blank liposomes (blk) used as a vehicle control were tested at 20 *μ*g/mL. One unit (U) of COX activity is the amount of enzyme that generates 1.0 *μ*mol of resorufin per minute at pH 8.0 and 25°C. Data are the mean from three independent experiments. Dunnett's multiple comparisons test using two-way ANOVA was performed: ^∗^*p* < 0.01, ^∗∗∗^*p* < 0.001, and ^∗∗∗∗^*p* < 0.0001 for free guttiferone E versus guttiferone E-loaded liposomes.

Cyclooxygenase (COX) is an enzyme responsible for the formation of important inflammatory mediators including prostaglandins, prostacyclin, and thromboxane. There are two known isoenzymes: COX-1 and COX-2. COX-1 is constitutively expressed in many tissues, while COX-2 is not expressed under normal conditions in most cells, but elevated levels are observed during inflammation. Inhibition of cyclooxygenase activity has been traditionally considered the most appropriate target for anti-inflammatory drugs [22]. In this study, the ability of guttiferone E-loaded liposomes to inhibit the activity of cyclooxygenase enzymes (COX-1 and COX-2) was determined by calculating their activity present in LPS-stimulated MH-S macrophage lysate. Cells were treated with different concentrations of guttiferone E-loaded liposomes and then stimulated with LPS; after 24 h incubation, the activity of COX-1 and COX-2 was measured and compared to the untreated control. Diclofenac, a nonsteroidal anti-inflammatory drug, an inhibitor of cyclooxygenase enzymes, was used as a reference. GE-liposomes significantly reduced the COX activity as compared to the untreated control. The anti-COX activity was concentration-dependent for both COX-1 and COX-2 (Figure 6). From Dunnett's multiple comparisons test using two-way ANOVA at *p* < 0.0001, no statistical difference was observed between the free guttiferone E and guttiferone E-loaded liposomes in inhibiting COX-1 or COX-2 activity.

When compared to the reference compound (diclofenac), the COX inhibitory activity of liposomes was weak. Considering the overall level of activities of GE-liposomes, COX-1 appeared to be more expressed than COX-2, but in turn, COX-1 activity was more inhibited than that of COX-2. The highest anti-COX activity was observed at 15 *μ*M of treatment, with 16.06 *μ*U and 14.53 *μ*U of COX activity detected, respectively, for COX-1 and COX-2. These values of activities corresponded, respectively, to 5.07-fold and 3.42-fold inhibition for COX-1 and COX-2, compared to untreated control which showed respective values of 81.51 *μ*U and 49.73 *μ*U of COX-1 and COX-2 activity detected.

## 4. Discussion

The significance of zeta potential is that its value can be related to the short- and long-term stability of emulsions; then, it can influence particle stability as well as cellular uptake and intracellular trafficking [23]. The encapsulation efficiency value indicated that 63.86% of the initial amount of guttiferone E was encapsulated. Although these results are interesting, optimization should be considered to increase the guttiferone E loading capacity of the liposomes. In this regard, the effect of various parameters on encapsulation efficiency could be studied. These include parameters such as a drug to phospholipid mass ratio or varying phospholipids. The incorporation of guttiferone E into DPPC/cholesterol liposomes could be demonstrated in this work. Important advances have been recently made showing that nanotechnology can significantly increase the pharmacological potential of natural products both in vitro and in vivo [23]. To the best of our knowledge, this is the first report describing a liposomal formulation for the delivery of the bioactive guttiferone E.

Drug screening for the identification of compounds with anticancer activity is commonly performed using various cell lines usually from different organs [24]. The general trend of the cytotoxicity for both guttiferone E-loaded liposomes and free guttiferone E was towards the reduction of cell viability. However, the extent of the cytotoxic effect varied depending on the cell line. This finding is not surprising taking into account the general complex relationship between the genetic features of the cell line and the drug response since each cell line has its genomic characteristics. A previous study reported that some drugs are effective against almost all types of cell lines, whereas certain drugs are effective against only a limited type of cell lines [25, 26]. According to Jaeger et al., differences in cancer susceptibility to a drug are due to interactions between inherited genetic factors which are constituted mainly by weakly acting low-penetrance genetic variants that interact among themselves. Therefore, several factors, beyond target expression, determine drug sensitivity [26]. Hence, these genomic features might explain the differences observed in the sensitivity of cell lines towards guttiferone E and guttiferone E-loaded liposomes. Although cancer cell growth inhibition was observed both for free guttiferone E and guttiferone E-loaded liposomes, the overall cytotoxic effect was more pronounced for guttiferone E alone as compared to that of GE liposomes. However, considering that free guttiferone E was here dissolved in DMSO, obtaining active guttiferone E-loaded liposomes in our study is a promising achievement considering that further optimization could be performed to improve its pharmacological expectations.

The results from the uptake studies indicate that GE liposomes are capable of being internalized into MH-S cells. The presence of GE liposomes labeled with rhodamine-DPPE inside the MH-S cells provides evidence that DPPC/CHO liposomes can be used for the delivery of the bioactive guttiferone E.

Liposomes as nanosized phospholipid-based vesicles are expected to solubilize guttiferone E and enhance its activity, but liposomal guttiferone E formulated in our work was found to be a little bit less potent than the corresponding free guttiferone E. However, it should be noted that free guttiferone E has been active only when dissolved in organic solvent or DMSO. In this work, guttiferone E was successfully encapsulated into liposomes, and thereby, the liposomes solubilize this compound, allowing a potential intravenous administration without the use of organic solvents. Formulated liposomes showed NO production inhibitory activity; this result is encouraging since the most suitable system with improved activity could be obtained by modifying the lipid composition.

COX inhibition studies showed that guttiferone E-loaded liposomes formulated have anti-inflammatory potential mediated by the cyclooxygenase pathway. Previous studies with bioactive natural products regarding anti-COX activity are very scarce. However, Coimbra et al. [27] reported that simple solubilization of poorly water-soluble natural compounds in liposomes can have important therapeutic benefits. In this work, poorly water-soluble guttiferone E was successfully solubilized in DPPC/CHO liposomes and still exhibited anti-inflammatory activity via nitric oxide production and cyclooxygenase activity inhibition.

## 5. Conclusions

In this study, we prepared a liposomal formulation with good entrapment efficiency of naturally occurring guttiferone E. The formulated liposomes selectively reduced the human cancer cell's viability and exerted a potent anti-inflammatory effect mediated by the nitric oxide production inhibition and cyclooxygenase pathway. Our result indicates that liposomes can be used as a carrier for the delivery of bioactive natural compounds. These findings open the possibility of the bioactive guttiferone E to move forward in the drug discovery pipeline for its future clinical use. However, further study with other phospholipids and another drug to phospholipid ratios should be considered to optimize the liposomal guttiferone E formulation.

## Figures and Tables

**Table 1:** IC_50_ values (*μ*g/mL) of free guttiferone E and guttiferone E-loaded liposomes against human cancer cell lines.

Cell lines	Guttiferone E-liposomes	Free guttiferone E	Doxorubicin
SKBr-3	22.25 ± 1.48^a^	12.28 ± 0.21^b^	0.98 ± 0.05^c^
SKOV-3	9.73 ± 0.54^a^	40.18 ± 1.67^b^	0.84 ± 0.08^c^
A549	17.55 ± 0.47^a^	9.79 ± 0.36^b^	1.15 ± 0.84^c^
Jurkat	10.40 ± 0.79^a^	5.46 ± 0.46^b^	0.61 ± 0.04^c^
CaCo-2	18.54 ± 1.31^a^	13.47 ± 0.05^b^	2.32 ± 1.04^c^
MH-S	22.43 ± 1.52^a^	21.87 ± 0.15^a^	1.84 ± 0.27^b^

Data represent the mean ± SD of three independent experiments. Within each row, values with different letters are significantly different at *p* < 0.0001, according to Dunnett's multiple comparisons test using two-way ANOVA.

## Data Availability

The data used to support the observations of this study are available from the corresponding author upon request.
